# Updating Standards of Facial Growth in Romanian Children and Adolescents Using the Anthropometric Method—A Pilot Study

**DOI:** 10.3390/ijerph18105288

**Published:** 2021-05-16

**Authors:** Emilia Ogodescu, Malina Popa, Magda Luca, Andreea Igna, Mariana Miron, Krisztina Martha, Anca Tudor, Carmen Todea

**Affiliations:** 1 Pediatric Dentistry Research Center, Department of Pediatric Dentistry, Faculty of Dentistry, “Victor Babes” University of Medicine and Pharmacy, Eftimie Murgu Square No. 2, 300041 Timisoara, Romania; luca.magda@umft.ro (M.L.); igna.andreea@umft.ro (A.I.); 2 Department of Oral Rehabilitation and Dental Emergencies, Faculty of Dentistry, “Victor Babes” University of Medicine and Pharmacy, Eftimie Murgu Square No. 2, 300041 Timisoara, Romania; miron.mariana@umft.ro (M.M.); todea.darinca@umft.ro (C.T.); 3 Department of Orthodontics, Faculty of Dentistry, “George Emil Palade” University of Medicine, Pharmacy, Science, and Technology of Târgu Mureș, Gheorghe Marinescu Street No. 38, 540142 Târgu Mureș, Romania; 4 Department of Functional Sciences, Faculty of Medicine, “Victor Babes” University of Medicine and Pharmacy, Eftimie Murgu Square No. 2, 300041 Timisoara, Romania; atudor@umft.ro

**Keywords:** facial growth, non-invasive measurements, anthropometry, reference values, healthy children and adolescents, orthodontic diagnosis, interceptive orthodontics

## Abstract

The anthropometric method is an important tri-dimensional and non-invasive assessment instrument for accurate diagnosis in paedodontics, orthodontics, and other medical fields. Our aim was to propose a technique that is accessible for clinicians and to determine the reference values for Romanian children and adolescents for the facial parameters selected. We proposed three basic instruments: a plastic compass, a ruler, and a digital caliper. Eighty-five children and adolescents (62% girls and 38% boys), aged between 3.5 and 14.5 years, were included in the study. We selected eight transversal, 12 vertical, and three sagittal measurements. Facial indices, according to Farkas L.G., were directly determined. The correlations between facial and general growth parameters, using Pearson correlation coefficient, for the entire sample were significant, direct, and strong for the following: Zy-Zy, Go-Go, N-Gn, N-Sn, Sto-Gn, Tr-Gn, Tr-Sn, Tr-Tr (r = 0.526–0.925, *p* < 0.001), and insignificant for Sn-Sto (r = 0.099–0.124, *p* > 0.354). The highest correlation coefficient is exhibited by Tr-Gn (r = 0.893 with height and r = 0.925 with weight). When performing a gender related comparison, we noticed that the vertical and transversal linear parameters and some facial indices are increased in boys (*p* < 0.05), depending on the age group. The simplified anthropometric technique represents an accessible method to every clinician, bringing important information related to dentofacial growth, diagnosis, and treatment planning in dentistry.

## 1. Introduction

Because dentists and, in particular, orthodontists, are involved in the development of not only the dentition, but the entire dentofacial complex, a practitioner may be able to manipulate the facial growth for the benefit of the patient. This fact requires a thorough understanding of both the pattern of normal growth and the mechanisms that underlie it [1]. In order to achieve the above-mentioned goal, dentists must have sufficient knowledge regarding the normal growth of the dentofacial complex and proper instruments represented by up-to-date standards.

Every person’s face is a custom-made original [2]. Enlow D.H. (1996) explained the biologic rationale underlying common variations in facial features in *Essentials of Facial Growth*, taking three general features into account: (1) different facial types as they relate to variations in the development of overall form and shape of the whole head, (2) gender related developmental facial differences, and (3) child and adult facial differences [2].

In 1930, Scammon described four growth curves for distinct tissues of the human body: the genital, the general, the neural, and the lymphoid growth curve [1,3]. Facial growth, including skeletal and soft-tissue growth, follows the general somatic growth; it respects the cranio-caudal growth principle [1,3]. Graber T.M. determined the quantity of the craniofacial growth for different age groups, expressed in percentages. In the 1–5-year group, the cranium increases by 85%, the maxilla by 45%, and the mandible by 40%; in the 5–10-year group, the cranium only modifies with 11%, the maxilla with 20% and the mandible with 25%. In the larger age interval ranging from 10 to 20 years, the cranium expends by only 4%, while the maxilla and mandible increase by 35% each [3,4].

In this context, it is important to know which are the values that practitioners should use as reference values and what is their meaning [5]. Our research started from the questions of how these standards were developed, and which are the values that practitioners use as standards. We tried to contribute to the development of reference values for Romanian growing population.

Craniofacial anthropometry is an objective technique, which is based on a series of linear and angular measurements and proportions; it facilitates the characterization of phenotypic variation and the evaluation of dysmorphology and dimensional variation during growth [6,7]. The assessment of craniofacial deformities by only visual examination is termed anthroposcopy; the method is highly subjective, and it does not provide quantitative data to the examiner [6,8]. The limits of anthropometry are represented by the sensitivity of some tissues to direct measurements, compressibility of soft tissues, differences of thicknesses and consistency of different tissues, and pressure of the instrument during measurement [9]. However, the method also has numerous advantages; it is a tri-dimensional technique that can deliver longitudinal data that are important for the orthodontic diagnosis and the craniofacial growth studies.

These direct facial measurements are effective and inexpensive, but difficult to perform, time consuming, and not conducive to a busy clinical practice. Recent advancements in technological field made the implementation of tri-dimensional photography or stereophotogrammetry possible, and a solution for the main disadvantages [10]. However, there is a lack of data for different age groups, until the new techniques can be widely used.

A recognized name in the field of craniofacial anthropometrics, Farkas L.G., who studied Canadian and Nordic European populations, mentioned that the craniofacial growth is predictable, however reference values should not be used for more than 20 years [5,11].

The craniofacial anthropometry can be used in many areas, such as the management of patients with cleft lip and palate (still lacking the reference values in infants), anaplasthology (possible to combine anthropometry and computer-aided design and manufacture technology for fabrication of epitheses), forensics (comparing recordings from video surveillance systems with manual measurements of the suspect subject), genetics and paediatrics (Down syndrome, plagiocephaly, craniosynostosis, and growth hormone deficit) for an early diagnosis of the anomalies and ergonomic product design [6,10,12,13,14,15,16]. Some authors determined an ameliorated facial profile due to improved mandibular development after treatment with growth hormone [17].

Proffit W. presented the commonly used measurement instruments (bow calipers and straight calipers) and pointed out the most important data of Farkas L.G. that were acquired by anthropometric measurements (linear and angular measurements, and facial indices) in young adults [1]. Clinicians need updated reference values for different populations, as determined on larger samples [18]. One study published in Canada (2007), compared the values between Americans of Caucasian origin and the Americans with African origin; significant differences in nasal and orbital region were recorded, which confirmed the necessity of reference values for each particular race [19].

The pattern and growth rate, the peripubertal growth spurt, and the differences between boys and girls were determined in a mixed longitudinal study, on individuals aged from six to 17 years, in Columbian mestizos; regional differences were recorded [20]. Anthropometric measurements for studies on facial growth have been also performed in Romania some decades ago by Firu P., Boboc G., Milicescu V., and Milicescu I.D. They used the measurements that were introduced by Rainer in 1937 and instruments developed by Martin and Firu [21,22,23].

Today, several competing methods are available for capturing and quantifying facial surface morphology, including two-dimensional photogrammetry. The recent development of facial tri-dimensional data acquisition (the accuracy and reliability using them being checked) reduced the time needed for assessment and increased the number of individuals of different age groups that can be included in studies [24,25,26,27,28,29,30,31,32,33,34,35,36]. Most of them were conducted on adults, highlighting the importance of our study on different age groups of Romanian children and adolescents using the traditional method.

The aims of our study were: (1) to implement simplified anthropometric instruments, accessible for every clinician and demonstrate the possibility of using this technique in growth studies; (2) to present the reference values obtained for selected facial parameters for each age group; (3) to compare the results between females and males and to other population; and, (4) to update the somatic growth general parameters at different ages and determine the possible correlation between them and the ones that characterize facial growth.

## 2. Materials and Methods

### 2.1. Instruments Used for the Simplified Orthodontic Technique in Orthodontics, Pedodontics and Growth Studies

**Figure 1:**
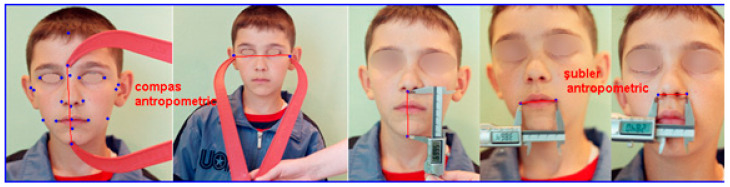
Specific instruments indicated for the simplified anthropometric method and examples of their use for the assessment of facial dimensions (plastic compass and digital caliper).

Because of the fact that achieving a complete set of anthropometric instruments or the devices that are needed for the tri-dimensional data acquisition is not possible in every country or in every dental clinic, we propose three basic instruments: (1) a plastic compass that has unsharp edges, (2) a ruler, which is up to 30 cm by mm graded, and (3) a digital caliper that is used for the study model analyses, with a slightly unsharp edges (Figure 1). The first two instruments are used together and preferred for every child, even for the ones who are noticeably young (3–7 years) or express some uncooperativeness. The third instrument is used for more precise measurements of the smaller dimensions, but it is not indicated in younger children because of the risk of damaging tegument or eyes. Besides of these instruments, we added a digital body weight scale and a height scale (composed of one horizontal rod that moves in vertical plane.

### 2.2. Selection and Marking of the Main Reference Points

**Figure 2:**
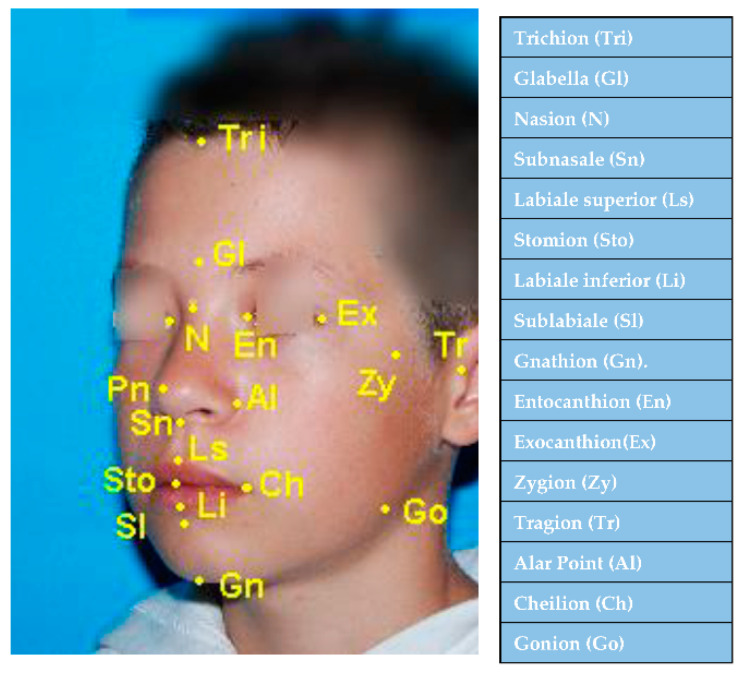
The median and lateral anthropometric points recommended for the anthropometric simplified technique.

We selected a number of eight transversal measurements, 12 vertical measurements, and three sagittal measurements, for which we presented standard values at different ages. We traced the cutaneous reference points (median, lateral and paramedian). The median reference points are: Trichion (Tri, Tr), Glabella (G, Gl), Nasion (N, Na), Subnasale (Sn), Labiale superior (Ls), Stomion (St, Sto), Labiale inferior (Li), Sublabiale (Sl) and Gnathion (Gn). The lateral reference points are: Zygion (Zy), Gonion (Go), Cheilion (Ch), Alar Point (Al), Entocanthion (En), Exocanthion (Ex), and Tragion (Tr, T). The point Cheilion (Ch) and the alar point (Al) are considered to be paramedian by some authors [37] (Figure 2).

### 2.3. The Workiflow on Children of Different Ages

**Figure 3:**
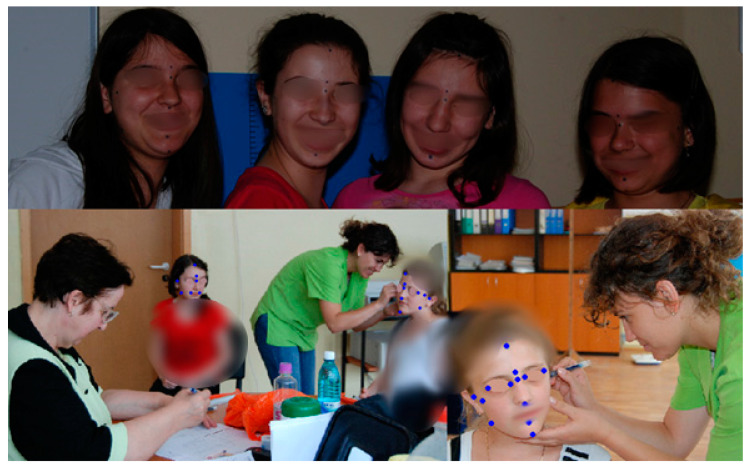
Marking of the median and lateral selected anthropometric reference points.

**Figure 4:**
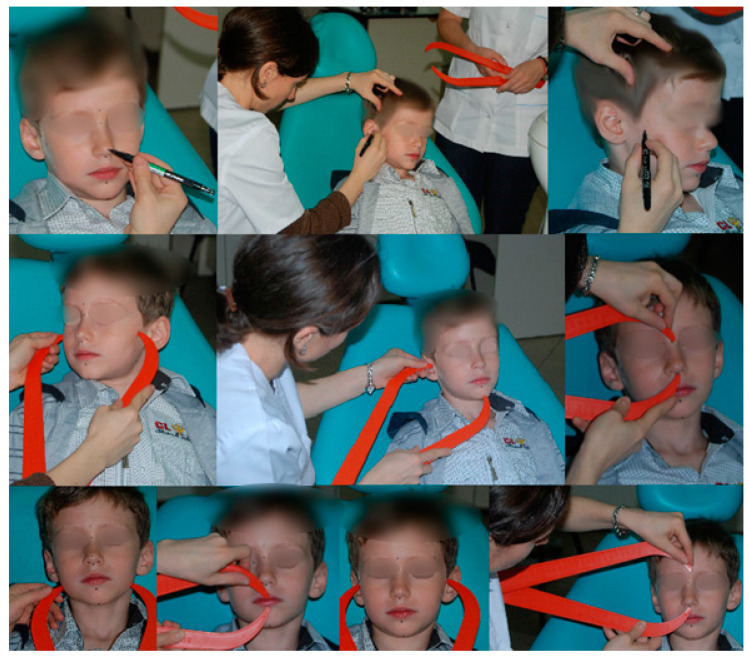
The simplified anthropometric technique used for the facial measurements.

The working flow includes two aspects: marking the reference points with a permanent marker or with an eyeliner and the measurement of the transversal, vertical, and sagittal parameters (Figure 3 and Figure 4).

One examiner and two assistants were involved in our study. One assistant has supervised the head position, with the Frankfurt horizontal plane being parallel to the floor, the correct location of landmarks, and the precision of measurements. The other assistant recorded the findings.

The selected transversal measurements are eight: Zygomatic width (maximal facial width; Zy-Zy); Gonial width (maximal mandibular width; Go-Go); Mouth width (maximal mouth width, measured from one mouth angle to the other; Ch-Ch), Nose width (maximal nose width; Al-Al); Nose basal width (En-En); the distance between the outer commissures of the eye fissures Ex-Ex); the distance between the N point and the middle of the left pupil; and, the width of the head between Tragus (Tr-Tr) [1,6,38].

The selected vertical measurements are 12, namely: Face height (N-Gn), upper face height (N-Sn), N-Sto, lower facial segment height (Sn-Gn), the height of both lower face segments, situated upper and lower to Sto (Sn-Sto and Sto-Gn), upper lip height (Sto-Ls) and lower lip height (Sto-Li), two segments of Sto-Gn, situated upper and lower to Sl (Sto-Sl and Sl-Gn), G-Sn, and Tri-Sn.

The selected sagittal measurements are three: superior facial depth (Tr-N), middle facial depth (Tr-Sn), and lower facial depth (Tr-Gn).

The values that were obtained by measurements, as done in the three spatial planes, were used for determining the reference values for different dimensions and different ages.

### 2.4. Sample Description, Study Protocol and Data Analysis

Our sample included 85 children and adolescents, from one nursery school and two schools, from Timisoara city and surroundings, Romania country. Fifty-three (62%) were girls and 32 boys (38%), aged between 3.5 and 14.5 years (Table 1). The sample was best represented for the groups that were included in 11.5–14.5-year intervals.

For the sample size calculation, we conducted a power analysis test with GPower3.1 application using the medium effect size, 0.05 level of significance, and 80% power.

The sample is representative, because it respects the proportions of age groups and categories according to BMI in the population (underweight 10.9%, normal weight 72.7%, overweight 10.9%, and obese 5.5%).

The time that is needed for each child measurement was 20 min., with the exception of the children aged between 3.5 and 6.5 years; in the last cases, the time was increased according to the ability to cooperate with each child.

Participation in the study was voluntary. Written informed consent was obtained from all of the the participants’ parents. The study has been carried out in accordance with the Declaration of Helsinki and it was approved by Institutional Ethics Committee (No 04/15.09.2008).

All of the measurements (expressed in mm) were transferred to an Excel Database. The facial indices, which were selected from the international literature (used by Farkas L.G.), were directly determined, according to the proportions that are presented in the literature [1,5,39].

The statistical analysis was performed using the SPSS version 17. Descriptive statistics were calculated for subjects’ characteristics (age, height, and weight) and facial parameters distributed in age groups (mean and standard deviation). We used the unpaired t test for comparing the independent values between sexes and linear correlation analysis using the Pearson correlation coefficient in order to determine the correlation between the facial and the general growth parameters. A *p* value of less than 0.05 was considered to be statistically significant.

The intra-examiner error has been calculated, by measuring a 25 children twice. The differences were non-significant, according to paired *t*-test (*p* > 0.05).

## 3. Results

Following the statistical analysis, we determined the mean, minimum, and maximum values for each measured facial parameter (Table 2).

The facial indices determined were: Facial Index (N-Gn/Zy-Zy); Mandible -facial width Index (Go-Go/Zy-Zy); Mandibular width-face height Index (Go-Go/N-Gn); Mandibular index or Jaw Index(Sto-Gn/Go-Go); Index of mouth width to jaw width (Ch-Ch/Go-Go); Index of upper lip height to mouth width (Sn-Sto/Ch-Ch); Index of lower lip height to mouth width (Sto-Sl/Ch-Ch); Upper Face Index (N-Sto/Zy-Zy); Lower face –face height Index (Sn-Gn/N-Gn); Mandible-face height Index (Sto-Gn/N-Gn); Mandible-upper face height Index (Sto-Gn/N-Sto); Mandible-lower face height Index(Sto-Gn/N-Sto); Index of upper to lower lip vermillion (Ls-Sto/Sto-Li); Index of upper to lower lip height (Sto-Sl/Sn-Sto); Index of upper lip to mandibular height (Sn-Sto/Sto-Gn); and, Index of Chin-face height (Sl-Gn/Sn-Gn) (Table 3).

**Figure 5:**
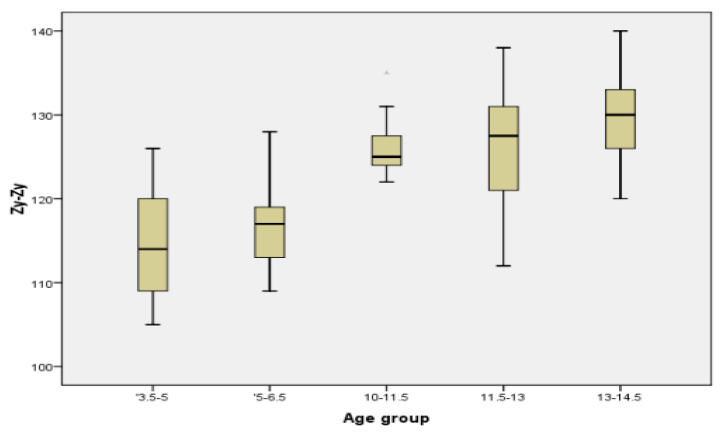
Boxplot for Zy-Zy values.

**Figure 6:**
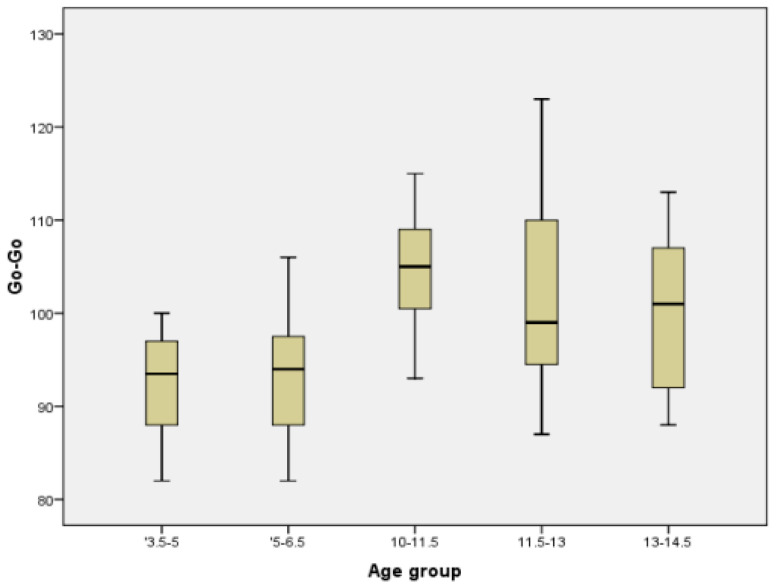
Boxplot for Go-Go values.

**Figure 7:**
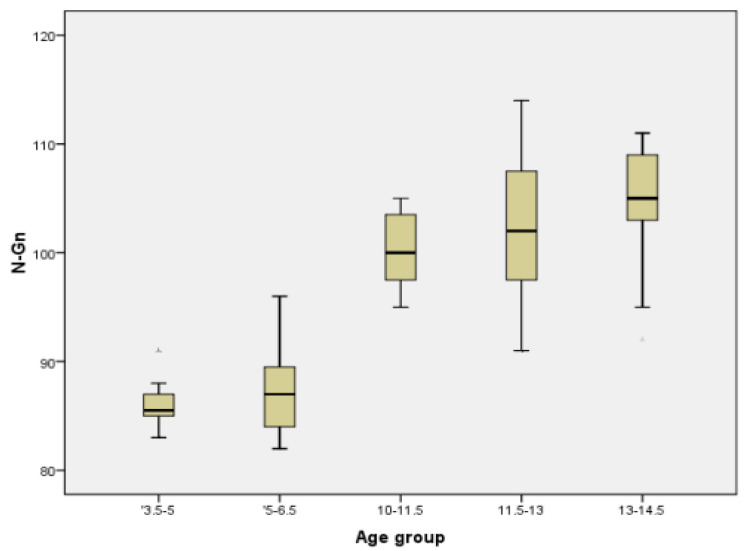
Boxplot for N-Gn values.

**Figure 8:**
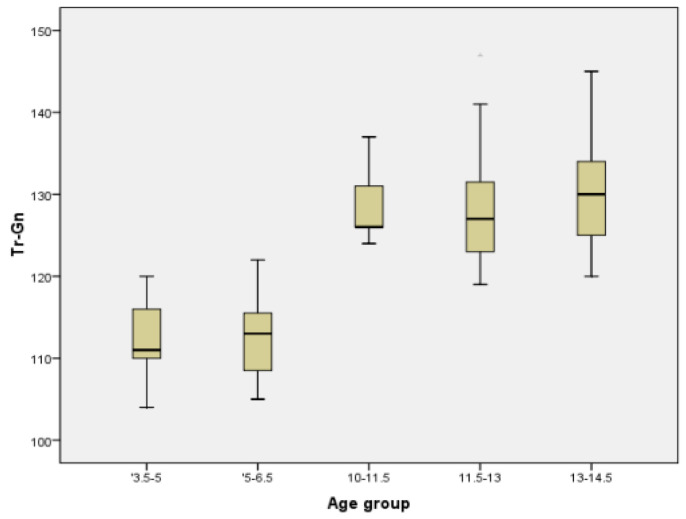
Boxplot for Tr-Gn values.

**Figure 9:**
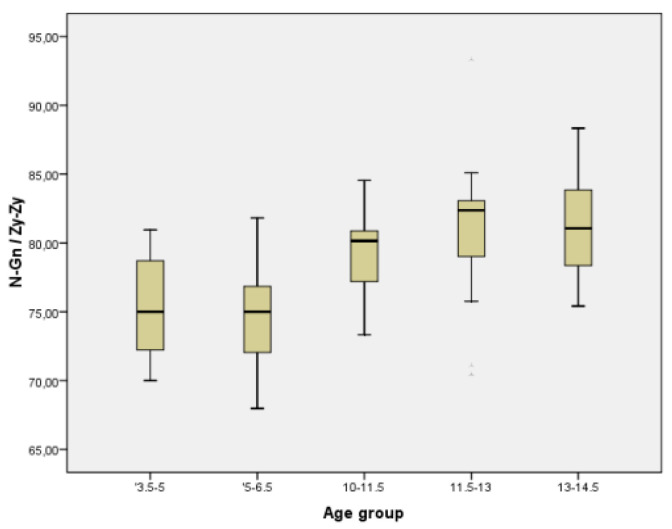
Boxplot for N-Gn/Zy-Zy values.

**Figure 10:**
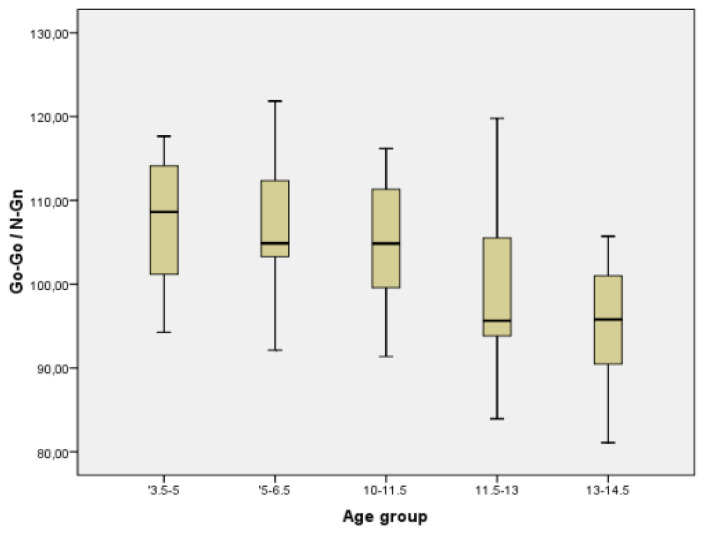
Boxplot for Go-Go/N-Gn values.

In order to have a better image regarding variation with age of different parameters, we chose two commonly measured transversal parameters (facial width and mandibular width), one vertical (facial height), one sagittal (Tr-Gn), and two facial indices (Facial Index and Mandibular width-Face Height Index) to be represented in the form of Boxplot (Figure 5, Figure 6, Figure 7, Figure 8, Figure 9 and Figure 10).

We determined the mean weight and height values, for one-year age intervals on a larger sample, including all of the values determined on children that participated in our dentofacial growth studies: the permanent teeth eruption study on a sample of 382 children (189 girls and 193 boys) aged between 3.5 and 15.5 years; the fotoanthropometric study on a sample of 156 children (55% girls and 45% boys) also aged between 3.5 and 15.5 years, and the present sample (Table 4).

We compared the values between boys and girls using the unpaired t test and taking the whole sample in account and determined significantly increased values for boys, regarding the following dimensions and facial indices: N-Gn (0.041^s^); N-Sn (0.031^s^); G-Sn (<0.001^s^); Tri-Sn (0.030^s^); N-Gn/Zy-Zy (0.026^s^); Go-Go/Zy-Zy (0.010^s^); Go-Go/N-Gn (0.002^s^); Sn-Gn/N-Gn (0.001^s^); and, Sto-Gn/N-Gn (0.049^s^). Other differences found were insignificant.

We also used the unpaired t test for the age group of 11.5–13 years (where the number of cases for boys and girls of our sample is consistent), for comparing the values between two sexes. The value for boys were significantly increased, for the following dimensions and facial indices: Zy-Zy (0.046^s^), Go-Go (0.007^s^), Ch-Ch (0.045^s^), Al-Al (0.049^s^), Sn-Gn (0.031^s^), Sn-Sto (0.001^s^), and Tr-Tr (0.004^s^). The other differences for the facial dimensions were insignificant. Four from all of the determined facial indices presented increased values for boys from this age group: Go-Go/N-Gn (0.029^s^), Sn-Sto/Ch-Ch (0.024^s^), Sn-Sto/Sto-Gn (0.030^s^), and Go-Go/Zy-Zy (0.010^s^).

The correlations between the facial parameters and values that were obtained for height and weight for the entire sample were significant, direct, and strong for the following dimensions: Zy-Zy, Go-Go, N-Gn, N-Sn, Sto-Gn, Tr-Gn, Tr-Sn, and sTr-Tr (r = 0.526–0.925, *p* < 0.001). There was an insignificant correlation for Sn-Sto (r = 0.099–0.124, *p* > 0.354). The higher correlation coefficient is exhibited by sagittal parameter Tr-Gn (r = 0.893 with height and r = 0.925 with weight) (Table 5).

## 4. Discussion

We conducted a cross-sectional study to characterize the facial growth by dividing the sample in five age intervals. We included two groups: the younger group (3.5–6.5 years) when considering the importance of early diagnosis for interception in this age interval and the older group (10–14.5 years) because of the importance of quantifying the growth at the age of puberty, when there is an increased somatic growth spurt and increased facial growth, and the initiation of dentofacial orthopaedic treatments according to the right assessment and diagnosis.

Calculating the body mass index (BMI) for each child, we obtained a distribution in the sample that is considered to be acceptable according to the variable percentage, regarding the distribution of BMI in Romanian children and adolescents.

Overweight and obesity vary between 17% and 30%, and they affect boys more than girls, particularly school age children than teenagers [40].

Another study determined that 8.29% of children are obese, while 12.84% of them are overweight, with boys being more likely to be obese; the highest prevalence has been observed among the 6–10 years’ age group, while teenagers have recorded the lowest prevalence [41].

In a study on children aged 6–19 years, across the last decade (2006–2015), which included Timisoara, the prevalence of underweight children was 5%/4.5%/8.5% (WHO/IOTF/CDC), while the prevalence of overweight (including obese) children was 28.3%/23%/23.2% (WHO/IOTF/CDC). This prevalence remained relatively stable over the last decade. Male gender, prepubertal age, and urban environment were the most relevant risk factors associated with overweight status in Romanian children [42].

Regarding the correlations among facial parameters and height and weight, there was one parameter (Sn-Sto) for which the correlation was insignificant, which suggested that the upper lip height has great interindividual variability. The strongest correlation with general growth was determined for Tr-Gn, which suggested the highest correlation with vertical and anterior mandibular growth.

When we started to assess the values of facial parameters at different ages and to determine the changes during the dentofacial growth, we should have begun by determining the reference values for the adults in that population. The values of facial dimensions in adults were published for the first time in 1981 in “Anthropometry of head and face in medicine; they were recommended by Profitt as up-to-date for the Caucasian population, and they are still currently used [1].

Non-invasive methods, like photographic and anthropometric, are preferred for both healthy subjects and patients, especially at young ages. After determining the reference values for our sample, we compared a part of the results with data from national literature (from some decades ago, published in 2001 and 2009) and from the international literature [1,21,23].

In Romania, Boboc G. determined the interzygomatic distance in a study on 100 adult crania. The recorded values were 131.1 mm (±5.54 mm) in men and 124.46 mm (±4.35 mm) in women. This means that, in adults, an important difference between sexes appears, when we take the facial width into account (7 mm larger in males). The cutaneous measurement values are higher with 10–15 mm than the osseous ones; the interzygomatic distance increases by 20 mm, starting from seven years to adulthood [23]. Proffit recommends smaller reference values for the interzygomatic distance, which makes direct facial measurements on young adults. This is 137 mm (±4.3 mm) in males and 130 mm (±5.3 mm) in females [1].

In our study, including the 3.5–14.5 years age interval, the mean interzygomatic distance (Zy-Zy) ranges between 115–129.5 mm. Boboc G. (in Romania) determined. in a sample (3–7 years age interval), a value raging between 110.3 and 118.93 mm for boys, and between 109 and 118.42 mm for girls. Milicescu (in Romania) determined (7–11 years age interval) a value between 125.5 mm and 129.6 mm for boys, and between 122.9 mm and 126.3 mm for girls [1,21,23].

The gonial width (Go-Go) that was determined by Farkas L.G. on adult population was 97 mm (±5.8 mm) mm for males and 91 mm (±5.9 mm) for females. Boboc (in Romania) determined on subjects included in 3–7 years age interval, a value of 80–87 mm for boys and 78–85 mm for girls. Milicescu (in Romania) determined (7–11 years age interval) a value between 98.3–98.4 mm for boys and 93.6–94 mm for girls. In our study, the mean value for the same parameter ranged between 92.5 mm and 104.5 mm, with an increased value when compared to those that were presented by Farkas, Milicescu, and Boboc [1,21,23]. Comparing our values with the values that were obtained by Milicescu, we noticed the decreased values between N-Gn (86–105 mm in our study, as compared to 101.5–108.5 mm for boys and 96.6–105.7 mm for girls), N-Sn (34–45.5 mm in our sample compared to 46.9–52.3 mm for boys and 42.4–50 mm for girls), and N-Sto (53–64.5 mm in our study when compared to 61.5–71.4 mm for boys and 65.6–68.9 mm for girls), as well as increased values for Tr-Tr (121–134.5 mm in our study as compared to 116.8–117.4 mm for boys and 113.3–114.1 mm for girls) in our sample [1,21].

Other mean values ranges for the whole sample of our study were then compared to the mean values of adults, as determined by Farkas, in order to have an overview of the results of our study. The mean value range for Ch-Ch in our study was 36–44 mm, compared to 53 mm for men and 50 mm for woman, as determined by Farkas. The smallest differences were assessed for parameters Al-Al (27.5–30.5 mm in our study, when compared to 35 mm for men and 31 mm for women), En-En (29.5–31 mm as compared to 33 mm for men and 32 mm for women) and N-middle of Pupilla (30–32 mm as compared to 33 mm for men and 31 mm for women). The determined lower face height (Sn-Gn) values in our study ranged between 51–60 mm, compared to those of Farkas (72 mm for men and 66 mm for women). We also compared the Upper lip vermillion and the Lower lip vermillion to the international reference values for adults (the determined Sto-Ls was 6–7.5 mm, as compared to 8.9 mm for men and 8.4 mm for women; the determined Sto-Li was 6.5–9 mm, when compared to 10.4 mm for men and 9.7 mm for women) [1].

The majority of data published in the international literature are for young adults. We compared the data of our study with reference values, for different population, with two of them being studied on children on 6–17 years age interval (Colombian mestizos) and 12–18 years age interval (boys from Nord of India) and the other two on Malaysian and Indo-Mauritian young adults, thus suggesting the existence of racial differences. [20,43,44,45]. Bizygomatic width for our sample ranged between 115 mm and 129.5 mm, whereas, for Indo-Mauritian young adults, it was 144 mm for men and 140 mm for women, 136.3 mm for Malaysian young adults, 120–136 mm for Colombian mestizos boys, and 118–131 mm for mestizos girls and only 99.45 mm for Nord Indian boys. The gonial width ranged in our sample between 92.5–104.5 mm, similar to 6–17 years’ girls of Colombian mestizos (93–104 mm), whereas boys presented 95–108 mm range values, 105 mm, respectively, 99 mm for Indo-Mauritian boys and girls and 83.5 for Nord Indian boys. The last-mentioned population had the smallest value. The determined Face height (N-Gn) in our sample was 86–105 mm, as compared to 94–117 mm and 92–109 mm for Colombian mestizos, boys, respectively, girls, 115.8 mm, respectively, 110 for Indo-Mauritian, men and women, 115.6 mm for Malaysian adults, and 102.4 for Indian boys. The determined upper face height (N-Sn) range was 34–45.5 mm, whereas it was 52 mm for Malaysian and Indo-Mauritian young adults and 47.8 for Indian boys. The determined Nose width (Al-Al, 29.5–31 mm) was compared to Malaysian (39.2 mm) and Indo-Mauritian (32.8 mm for men and 29.5 mm for women) young adult values. We only compared the different parameter values from our sample with Indo-Mauritian young adults’ values because of a lack of results in other studies (36–44 mm as compared with 47.9 for mouth width; 29.5–31 mm compared with 33.2 mm for nose basal width; 51–60 mm when compared with 65.9 mm for lower face height; 6–7.5 mm compared with 9.4 for upper lip vermillion; and, 6.5–9 mm compared with 11.5 mm for lower lip vermillion). We could not compare the N-middle of Pupilla and Tr-Tr because of a lack of values for these parameters in these studies [20,43,44,45].

The facial indices were compared to the values that were determined by Farkas (1987) in adults and in 4/5-year children (2003). In our study, for the 3.5–5-year-group (as compared to 4/5-year sample of Farkas), we noticed the following: the Facial Index (N-Gn/Zy-Zy) is decreased (75.34 ± 3.87 compared to 87.6 ± 4.2/86.7 ± 3.6 for boys and 86.8 ± 4.9/88.3 ± 4.8 for girls); the Mandibular Index (Sto-Gn/Go-Go) is decreased (38.7 ± 4.21 compared to 47.9 ± 4.9/48.5 ± 3.2 for boys and 48.3 ± 4.7/48.3 ± 3.8 for girls); the index of mouth width to jaw width (Ch-Ch/Go-Go) is decreased (38.77 ± 2.66 compared to 45 ± 3.9/46.7 ± 2.4 for boys and 46.7 ± 7.4 /46.2 ± 3.1 for girls); the index of upper lip height to mouth width (Sn-Sto/Ch-Ch) is decreased (48.77 ± 5.79 compared to 50.2 ± 4.8 /48.1 ± 4.3 for boys and 49.4 ± 4.8/48.0 ± 5 for girls); the index of lower lip height to mouth width (Sto-Sl/Ch-Ch) is increased (41.4 ± 5.71 when compared to 38.8 ± 4.5/37.5 ± 3.4 for boys and 38.1 ± 4.4 /38.4 ± 3.3 for girls); the index of upper to lower lip vermillion (Ls-Sto/Sto-Li) is decreased (79.99 ± 19.42 compared to 110.6 ± 25.2 /103.9 ± 17.3 for boys and 113.0 ± 23.7 /107.3 ± 21.9 for girls).; the index of upper to lower lip height (Sto-Sl/Sn-Sto) is increased (85.5 ± 12 compared to 77.7 ± 6.1/78.3 ± 5.9 for boys and 77.7 ± 8.8/80.5 ± 7.3 for girls); and, the index of upper lip to mandibular height (Sn-Sto/Sto-Gn) is increased (49.08 ± 6.39 as compared to 47.7 ± 5.4 /46.3 ± 3.9 for boys and 46.6 ± 4.3/45.8 ± 3.3 for boys). The mean values ranges for the whole sample of our study were then compared to the mean values of adults, as determined by Farkas, in order to have an overview of the results of our study (the determined Facial Index (N-Gn/Zy-Zy) range was 75.3–81.3, as compared to 88.5 ± 5.1 in adult men and 86.2 ± 4.6 in adult women, showing that this increases with age and the quantity of increase to adulthood; the Mandible-Face width (Go-Go/Zy-Zy) range was 77.2–82.7, compared to 70.8 ± 3.8 for adult men and 70.1 ± 4.2 for adult females, presenting a decrease with age; the Mandibular width-Face Height (Go-Go/N-Gn) range was 95.2–107.48, compared to 80.3 ± 6.8 for adult men and 81.7 ± 6.0 for adult women, demonstrating a higher decrease with age; the Upper face Index (N-Sto/Zy-Zy) range was 46.13–50.62, compared to 54 ± 3.1 for adult men and 52.4 ± 3.1 for adult women, showing an slight increase with age; determined Mandibular Index (Sto-Gn/Go-Go) range was 35.36–41.01 compared to 51.8 ± 6.2 for adult men and 49.8 ± 4.8 for adult women, suggesting a consistent increase with age; and, the Mandible-Face Height range was (Sto-Gn/N-Gn) range was 36.77–41.38, compared to 41.2 ± 2.3 for adult male and 40.4 ± 2.1 for adult female, which suggests that this proportion only suffers a slight increase to adulthood). [1,5].

### 4.1. Study Limitations

We noticed that most of the values (in mm), for the determined dimensions, increase with age, except for certain age groups, for which they are similar or even decrease. The small sample size, when divided to age groups and the increased age intervals (1.5 years) is a possible explanation. We had insufficient data in small age groups (3.5–5 and 5–6.5) to determine En-/En and N-middle of the left pupil, due to the difficult cooperation of small children. The comparison to other anthropometric studies was difficult, as the samples have a different structure of age intervals and the used values were the mean values, which were determined for the whole sample. It suggests the need for international and interregional studies, on the same age subdivision of the sample (six months–one year), using a standardized study protocol and the same digital instruments for measurement.

Considering that, today, some powerful tools for anthropometric tridimensional analysis are available free of charge or at a limited cost, the potential impact of the method that was proposed in this study is limited. Several smartphones are able to capture three-dimensional facial images, which can be analysed using freeware software, such as Viewbox (Viewbox 4, dHAL software, Kifissia, Greece), for geometric morphometric analysis [29].

### 4.2. Future Perspectives

There is a high necessity for anthropometric studies on larger samples of healthy subjects of different ages: new-borns, children in 4 to 10-year age interval, children in 10 to 15–16-year- age interval (around the peripubertal growth spurt), and young adults. The samples should be divided in six months or one year age subgroups, with a higher number of individuals for each subgroup and a balanced gender distribution. The reference values can be obtained from transversal growth studies on large samples or from longitudinal or mixed longitudinal studies.

The future growth studies should include the children and their parents in order to determine whether there are correlations between dimensions and proportions of family members; this could provide new reference values for children and adults at the same time. Three main measurements (Zy-Zy, Go-Go, and N-Gn) and two main facial indices (Facial Index and Mandibular width-face height Index) should be included in order to have an accessible assessment method, giving the possibility to analyse large samples, including transversal and longitudinal data.

Correlations between the recorded values using direct anthropometric measurements, parameters obtained by using standardized and calibrated digital photography analysed with special software, and measurements taken from 3D surfaces generated with stereophotogammetry are necessary to improve the workflow and applicability in different medical fields.

The development of interregional “Centers for dentofacial growth and development studies” also represents a need for the future perspectives.

## 5. Conclusions

In our study on healthy children and adolescents, the main anthropometric facial parameters (N-Gn, Zy-Zy, and Go-Go) increased with age. The relationship between them (as expressed by three facial indices) suffered the following changes: facial index increased with age demonstrating an increased change of the dimension N-Gn, when comparing to Zy-Zy; mandible-facial width index decreased demonstrating an accentuated growth of Zy-Zy comparing to Go-Go; mandibular width –face height index decreased with age, and it is related to higher dimensional change of parameter N-Gn, when compared to Go-Go. The N-Gn parameter has the highest rate of change, followed by Zy-Zy, and then by Go-Go. Some facial parameters suffer the small dimensional changes during growth and development (En-En, Al-Al, N-middle of Pupilla and Sn-Sto). The Nose Length (N-Sn), the Upper and middle figure height (Tri-Sn), and the sagittal parameters (Tr-Gn, Tr-Sn, and Tr-N) change significantly with age. When performing a gender related comparison, we noticed that the vertical and transversal linear parameters and some facial indices are increased in boys, depending on the age group. The correlations between facial and general growth parameters are significant in both girls and boys, except for one dimension (Sn-Sto).

We conclude that the simplified anthropometric technique represents an accessible method to every clinician, bringing important information that is related to dentofacial growth, diagnosis, and treatment planning in dentistry.

## Figures and Tables

**Table 1:** Age-groups distribution of the study subjects.

Age-Group (Years)	Number of Subjects	Percentage (%)
3.5–5	14	16.5%
5–6.5	11	13%
10–11.5	11	13%
11.5–13	28	33%
13–14.5	21	24.5%

**Table 2:** Facial dimensions in our study on different age groups.

Facial Dimensions (mm)	3.5–5 Years Mean ± SD (Min–Max)	5–6.5 Years Mean ± SD (Min–Max)	10–11.5 Years Mean ± SD (Min–Max)	11.5–13 Years Mean ± SD (Min–Max)	13–14.5 Years Mean ± SD (Min–Max)
Zy-Zy	114.79 ± 6.15 (105–126)	116.91 ± 6.02 (109–128)	126.18 ± 3.87 (122–135)	126.25 ± 6.52 (112–138)	129.43 ± 5.48 (120–140)
Go-Go	92.64 ± 5.61 (82–100)	93.45 ± 7.33 (82–106)	104.45 ± 6.9 (93–115)	101.18 ± 9.67 (87–123)	100.1 ± 7.71 (88–113)
Ch-Ch	35.93 ± 3.43 (31–42)	36.82 ± 2.86 (32–41)	43.09 ± 3.39 (37–48)	44.36 ± 3.72 (35–51)	44.14 ± 3.93 (36–54)
Al-Al	27.43 ± 1.6 (26–32)	27.27 ± 1.56 (26–31)	30.36 ± 2.01 (26–33)	29.64 ± 2.77 (25–37)	30.29 ± 2.08 (26–33)
En-En	-	-	29.55 ± 2.21 (25–33)	30.36 ± 2.2 (26–37)	31.33 ± 2.39 (25–37)
N-middle of Pupilla	-		29.91 ± 0.83 (29–31)	29.86 ± 2.74 (24–35)	31.9 ± 1.87 (28–35)
Ex-Ex	91.21 ± 6.64 (80–102)	96.18 ± 6.42 (87–106)	99.25 ± 4.86 (90–108)	97.36 ± 5.1 (89–109)	103.5 ± 4.95 (100–107)
N-Gn	86.29 ± 2.37 (83–91)	87.55 ± 4.32 (82–96)	100.09 ± 3.48 (95–105)	102.25 ± 6.22 (91–114)	105.19 ± 5 (92–111)
N-Sn	34.29 ± 1.73 (31–37)	36.09 ± 3.81 (31–43)	45.45 ± 2.25 (42–49)	43.68 ± 3.54 (38–50)	44.62 ± 3.83 (36–52)
N-Sto	52.86 ± 2.57 (49–57)	55.36 ± 3.07 (51–60)	63.82 ± 3.09 (58–68)	62.96 ± 4.35 (54–71)	64.43 ± 3.11 (60–71)
Sn-Gn	51.29 ± 2.16 (49–57)	52.18 ± 3.09 (46–57)	57.18 ± 5.13 (52–68)	58.61 ± 4.43 (50–66)	60 ± 4.1 (52–66)
Sn-Sto	17.43 ± 1.87 (14–20)	19.55 ± 4.34 (12–29)	19.09 ± 3.21 (14–25)	18.68 ± 2.83 (12–23)	18.9 ± 1.41 (16–21)
Sto-Gn	35.71 ± 3.02 (30–40)	34.45 ± 1.21 (33–37)	36.82 ± 3.79 (31–43)	39.96 ± 3.14 (32–45)	40.86 ± 3.35 (33–48)
Sto-Ls	6.25 ± 1.55 (3–9)	6.41 ± 1.71 (4–10)	7.55 ± 1.04 (6–9)	6.89 ± 1.49 (4.5–11)	6.67 ± 1.56 (4–10)
Sto-Li	7.89 ± 1.39 (5–10)	6.45 ± 1.13 (5–9)	8.91 ± 1.76 (6–11)	9.07 ± 1.22 (6.5–12)	8.9 ± 1.79 (7–13)
Sto-Sl	14.79 ± 1.76 (12–18)	13.36 ± 1.91 (11–16)	16.18 ± 2.44 (11–18)	17.02 ± 1.99 (13–22)	16.29 ± 2.28 (13–21)
Sl-Gn	20.93 ± 3.41 (13–25)	21.09 ± 2.77 (17–26)	20.64 ± 2.46 (18–25)	23.55 ± 4.07 (16–35)	24.57 ± 3.16 (17–30)
G-Sn	42.79 ± 2.46 (40–47)	44.27 ± 3.93 (38–50)	59.09 ± 4.53 (52–69)	53.75 ± 4.97 (47–69)	55 ± 3.41 (48–61)
Tri-Sn	95.71 ± 4.1 (91–103)	97 ± 6.07 (89–107)	105.45 ± 4.61 (101–114)	107.86 ± 8.98 (90–122)	113 ± 8.83 (97–128)
Tr-Gn	112.14 ± 4.85 (104–120)	112.55 ± 5.35 (105–122)	128.45 ± 4.2 (124–137)	128.36 ± 6.93 (119–147)	130 ± 6.38 (120–145)
Tr-Sn	103.57 ± 2.24 (99–107)	103.45 ± 6.02 (94–111)	115.36 ± 3.32 (111–122)	115.57 ± 5.33 (108–131)	118.14 ± 6.24 (105–127)
Tr-N	100.86 ± 2.68 (94–104)	101 ± 3.44 (95–105)	110.55 ± 3.53 (106–119)	112.54 ± 5.1 (102–125)	114.38 ± 4.75 (107–121)
Tr-Tr	121.21 ± 4.37 (112–126)	122.36 ± 5.1 (115–131)	130.64 ± 4.84 (121–137)	132.25 ± 6 (120–144)	134.38 ± 6.36 (122–145)

SD: standard deviation.

**Table 3:** Facial anthropometric indices in our study on different age groups.

Facial Anthropometric Indices	3.5–5 Years Mean ± SD (Min–Max)	5–6.5 Years Mean ± SD (Min–Max)	10–11.5 Years Mean ± SD (Min–Max)	11.5–13 Years Mean ± SD (Min–Max)	13–14.5 years Mean ± SD (Min–Max)
N-Gn/Zy-Zy	75.34 ± 3.87 (70–80.95)	75 ± 4.25 (67.97–81.82)	79.38 ± 3.49 (73.33–84.55)	81.07 ± 4.46 (70.45–93.33)	81.33 ± 3.55 (75.41–88.33)
Go-Go/Zy-Zy	80.84 ± 5.32 (69.84–91.67)	79.88 ± 3.52 (75.23–85.45)	82.77 ± 4.59 (75–88.8)	80.03 ± 4.71 (72–89.15)	77.26 ± 3.43 (70.31–82.31)
Go-Go/N-Gn	107.48 ± 7.72 (94.25–117.65)	106.83 ± 7.81 (92.13–121.84)	104.51 ± 8.27 (91.35–116.16)	99.07 ± 8.84 (83.93–119.79)	95.24 ± 6.95 (81.08–105.71)
Sto-Gn/Go-Go	38.7 ± 4.21 (31.96–46.34)	37.1 ± 3.54 (31.13–43.9)	35.36 ± 4.19 (30.1–45.26)	39.83 ± 4.66 (28.57–47.13)	41.01 ± 4.34 (34.02–51.14)
Ch-Ch/Go-Go	38.77 ± 2.66 (33.7–42.42)	39.47 ± 2.61 (36.79–44.09)	41.33 ± 3.23 (35.92–46.94)	44.04 ± 3.87 (36.52–52.22)	44.19 ± 3.48 (38.53–53.47)
Sn-Sto/Ch-Ch	48.77 ± 5.79 (39.02–60.61)	52.78 ± 9.1 (37.5–70.73)	44.88 ± 10.2 (29.17–67.57)	42.23 ± 6.15 (26.67–51.11)	43.11 ± 4.7 (33.33–55.56)
Sto-Sl/Ch-Ch	41.4 ± 5.71 (32.43–54.84)	36.27 ± 4.05 (29.73–41.03)	37.74 ± 6.4 (25–46.15)	37.19 ± 8.95 (0–52.38)	37.17 ± 6.45 (27.66–58.33)
N-Sto/Zy-Zy	46.13 ± 2.58 (42.86–51.85)	47.44 ± 3.2 (42.97–52.29)	50.62 ± 2.87 (45.19–53.66)	49.93 ± 3.3 (42.64–55.47)	49.83 ± 2.51 (45.26–55.47)
Sn-Gn/N-Gn	59.44 ± 1.87 (56.98–62.64)	59.63 ± 2.72 (56.1–63.22)	57.08 ± 3.96 (53–66.02)	57.34 ± 3.18 (52.78–65.66)	57.04 ± 2.79 (51.46–61.9)
Sto-Gn/N-Gn	41.38 ± 3.22 (34.88–46.51)	39.44 ± 2.22 (35.42–42.53)	36.77 ± 3.33 (31–41.35)	39.14 ± 2.95 (32.32–44.9)	38.86 ± 2.91 (33.02–44.44)
Sto-Gn/N-Sto	67.68 ± 6.15 (56.6–77.55)	62.41 ± 4.11 (56.67–68.63)	57.73 ± 5.54 (48.44–65.15)	63.61 ± 4.91 (52.24–74.58)	63.58 ± 6.37 (51.47–78.69)
Sto-Gn/Sn-Gn	69.68 ± 5.79 (59.62–81.63)	66.24 ± 4.52 (59.65–72)	64.49 ± 5.12 (58.49–73.08)	68.33 ± 4.78 (59.26–78.57)	68.23 ± 5.56 (61.11–84.91)
Ls-Sto/Sto-Li	79.99 ± 19.42 (42.86–120)	100.88 ± 27.62 (66.67–150)	86.41 ± 12.94 (70–114.29)	76.19 ± 14.09 (55.56–122.22)	75.52 ± 14.32 (50–100)
Sto-Sl/Sn-Sto	85.5 ± 12 (70–106.25)	70.76 ± 15.55 (50–100)	86.67 ± 19.08 (61.11–121.43)	89.87 ± 22.92 (0–133.33)	86.68 ± 14.51 (66.67–125)
Sn-Sto/Sto-Gn	49.08 ± 6.39 (38.89–64.52)	56.85 ± 13.1 (34.29–85.29)	52.19 ± 9.23 (36.84–69.44)	46.93 ± 7.5 (27.27–60)	46.55 ± 4.79 (33.33–54.55)
Sl-Gn/Sn-Gn	40.76 ± 6.24 (26.53–48.98)	40.58 ± 6.04 (30.91–50)	36.13 ± 3.31 (32.14–41.67)	40.28 ± 7.07 (29.63–61.4)	40.93 ± 4.4 (31.48–50.85)

SD: standard deviation.

**Table 4:** Age group distribution of the general growth parameters in study subjects.

Age Group (Years)	Girls		Boys	
	Height (cm) (Mean ± SD)	Weight (kg) (Mean ± SD)	Height (cm) (Mean ± SD)	Weight (kg) (Mean ± SD)
3.5–4.4	107 ± 4.97	18.21 ± 2.18	106.67 ± 4.93	17.67 ± 2.52
4.5–5.4	110 ± 5.77	20.42 ± 3.77	112.86 ± 6.41	21.54 ± 4.07
5.5–6.4	118.75 ± 3.69	22.64 ± 2.55	126 ± 7.79	24.71 ± 4.93
6.5–7.4	122.67 ± 6.43	22.75 ± 1.71	124.75 ± 6.58	25.41 ± 5.34
7.5–8.4	132.67 ± 4.95	33.21 ± 8.52	132.57 ± 7.39	33.57 ± 9.84
8.5–9.4	133.18 ± 5.91	33.58 ± 7.17	135.39 ± 8.77	31.29 ± 5.52
9.5–10.4	139.27 ± 5.7	33.24 ± 5.94	133.29 ± 12.11	33.65 ± 6.52
10.5–11.4	149.83 ± 8.35	43.91 ± 12.92	147.33 ± 6.41	39.92 ± 7.86
11.5–12.4	154.56 ± 8.25	45.01 ± 9.74	152.74 ± 7.09	46.85 ± 10.29
12.5–13.4	157.47 ± 6.71	45.32 ± 7.28	161.33 ± 9.45	53.17 ± 12.49
13.5–14.4	161.08 ± 5.3	49.78 ± 8.06	164.25 ± 5.28	58.6 ± 9.72
14.5–15.4	159.5 ± 2.12	53.75 ± 9.55	165.83 ± 11.58	58.43 ± 21.93

SD: standard deviation.

**Table 5:** The correlations between facial parameters and general growth parameters.

Facial Parameters	Height		Weight	
	Correlation Coefficient	*p*-Value	Correlation Coefficient	*p*-Value
Zy-Zy	0.827	<0.001	0.805	<0.001
Go-Go	0.526	<0.001	0.626	<0.001
N-Gn	0.938	<0.001	0.895	<0.001
N-Sn	0.847	<0.001	0.751	<0.001
Sn-Sto	0.124	0.354	0.099	0.444
Sto-Gn	0.783	<0.001	0.785	<0.001
Tr-Gn	0.893	<0.001	0.925	<0.001
Tr-Sn	0.861	<0.001	0.848	<0.001
Tr-Tr	0.814	<0.001	0.720	<0.001

## Data Availability

The data presented in this study are available on request from the corresponding author.
